# Comparative Proteomic Analysis of *Methanothermobacter themautotrophicus* ΔH in Pure Culture and in Co-Culture with a Butyrate-Oxidizing Bacterium

**DOI:** 10.1371/journal.pone.0024309

**Published:** 2011-08-31

**Authors:** Miho Enoki, Naoya Shinzato, Hiroaki Sato, Kohei Nakamura, Yoichi Kamagata

**Affiliations:** 1 Research Institute of Biological Resources, National Institute of Advanced Industrial Science and Technology (AIST), Tsukuba, Japan; 2 Marine Biotechnology Institute, Kamaishi, Iwate, Japan; 3 Research Institute for Environmental Management Technology, National Institute of Advanced Industrial Science and Technology (AIST), Japan; 4 Research Institute of Genome-Based Biofactory, National Institute of Advanced Industrial Science and Technology (AIST), Toyohira-ku, Sapporo, Japan; 5 Tropical Biosphere Research Center, University of the Ryukyus, Nishihara-cho, Okinawa, Japan; 6 Faculty of Applied Biological Sciences, Gifu University, Gifu, Gifu, Japan; Auburn University, United States of America

## Abstract

To understand the physiological basis of methanogenic archaea living on interspecies H_2_ transfer, the protein expression of a hydrogenotrophic methanogen, *Methanothermobacter thermautotrophicus* strain ΔH, was investigated in both pure culture and syntrophic coculture with an anaerobic butyrate oxidizer *Syntrophothermus lipocalidus* strain TGB-C1 as an H_2_ supplier. Comparative proteomic analysis showed that global protein expression of methanogen cells in the model coculture was substantially different from that of pure cultured cells. In brief, in syntrophic coculture, although methanogenesis-driven energy generation appeared to be maintained by shifting the pathway to the alternative methyl coenzyme M reductase isozyme I and cofactor F_420_-dependent process, the machinery proteins involved in carbon fixation, amino acid synthesis, and RNA/DNA metabolisms tended to be down-regulated, indicating restrained cell growth rather than vigorous proliferation. In addition, our proteome analysis revealed that α subunits of proteasome were differentially acetylated between the two culture conditions. Since the relevant modification has been suspected to regulate proteolytic activity of the proteasome, the global protein turnover rate could be controlled under syntrophic growth conditions. To our knowledge, the present study is the first report on N-acetylation of proteasome subunits in methanogenic archaea. These results clearly indicated that physiological adaptation of hydrogenotrophic methanogens to syntrophic growth is more complicated than that of hitherto proposed.

## Introduction

Methanogenic archaea are generally found in anoxic environments such as aquatic sediments, anaerobic sewage reactors, and animal intestines, where complex organic matters are degraded in a step-wise process by some types of anaerobic microorganisms and finally converted into methane and CO_2_. In anoxic environments, low-molecular-weight fatty acids such as butyrate, propionate, and acetate are difficult to degrade because the anaerobic oxidation of these compounds is energetically unfavorable unless H_2_ partial pressure is kept very low. These processes are progressed by the association between fatty acid-oxidizing H_2_-producing syntrophic bacteria and H_2_-scavenging microbes such as hydrogenotrophic methanogens, which are underpinned by interspecies H_2_ transfer [1]. In this respect, mutualistic associations are established between syntrophs and hydrogenotrophic methanogens, and they are indispensable for complete oxidation of organic matter in methanogenic ecosystems.

In natural ecosystems, hydrogenotrophic methanogens live on a scarce amount of H_2_ provided by a syntrophic partner, which is at least three orders of magnitude lower than that provided for ordinary laboratory pure cultures (10^5^ Pa). Since H_2_ concentrations in syntrophic coculture are expected to be kept significantly low during growth, it is technologically difficult to mimic the syntrophic growth of methanogens using chemostat culture. Hence, almost nothing is known about the physiology of methanogens under syntrophic conditions and how methanogens have adapted to such H_2_-limitted natural environments.

We have had a long-term interest in the physiology of hydrogenotrophic methanogens under syntrophic association, and we have analyzed gene and protein expressions of *Methanothermobacter thermautotrophicus* strain TM using cells grown in pure culture and with an acetate-oxidizing syntroph, *Thermacetogenium phaeum* strain PB [2]. The results revealed that both gene and protein expressions of methyl coenzyme M reductase isozymes (MCRI and II), which are the key enzymes for methanogenesis, were significantly different from each other. In other words, methanogen cells under syntrophic conditions preferentially utilized MCRI, whereas pure cultured cells expressed both isozymes equally. Many studies on the changes in MCR isozyme expression using chemostat cultures evidenced that they are strictly regulated by H_2_ availability of the methanogen cells [3].

In this respect, a preferential use of MCRI implicated that H_2_-limitation has been believed to be a major factor characterizing physiological status of the syntrophically grown methanogens. However, recently, *M. thermautotrophicus* strain ΔH was reported to make aggregations with syntrophic bacteria via pili-like structures stretching from the syntrophic partners, conferring more efficient H_2_ transfer [4]. Such close cell interaction may accompany an unknown physiological response that is characteristic of the syntrophic growth of the two organisms.

To better understand the physiological features under syntrophic associations that occur in natural environments, detailed, comprehensive studies of gene and protein expressions must be examined. For this purpose, two-dimensional gel electrophoresis (2-DE) is a powerful method to display total protein expression and provide information on protein features, such as post- and cotranslational modifications, and it has already been used to describe the physiology of various microbes [5], [6], [7], [8].

In the present study, we conducted a comparative proteome analysis of *M. thermautotrophicus* ΔH cells in pure culture conditions with abundant H_2_ supply as well as in syntrophic (coculture) conditions with poor H_2_ supply to clarify the physiological states of hydrogenotrophic methanogens living on interspecies H_2_ transfer. The results showed that in addition to commonly studied MCR isozymes, there are a substantial number of other differentially expressed proteins that belong to a variety of functional categories. Involvement of these proteins in syntrophic growth was previously not known and indicated that physiological adaptation of methanogens to syntorphic growth is more complicated than that of hitherto proposed.

## Results

### Growth of methanogen

To investigate the physiology of hydrogenotrophic methanogens living on interspecies H_2_ transfer, we constructed a model coculture consisting of a hydrogenotrophic methanogen, *M. thermautotrophicus* ΔH, and an H_2_-producing butyrate oxidizer, *S. lipocalidus* TGB-C1. In this coculture, *S. lipocalidus* converts butyrate to acetate, H_2_, and CO_2_. The latter two are subsequently provided to *M. thermautotrophicus* as substrates for methanogenesis, in which interspecies H_2_ transfer underpins their growth and energy conservation. Growth of *M. thermautotrophicus* in the coculture depends on butyrate degradation rate and the efficiency of interspecies H_2_ transfer; hence, it was quite slow compared to growth in pure culture conditions.

**Figure 1:**
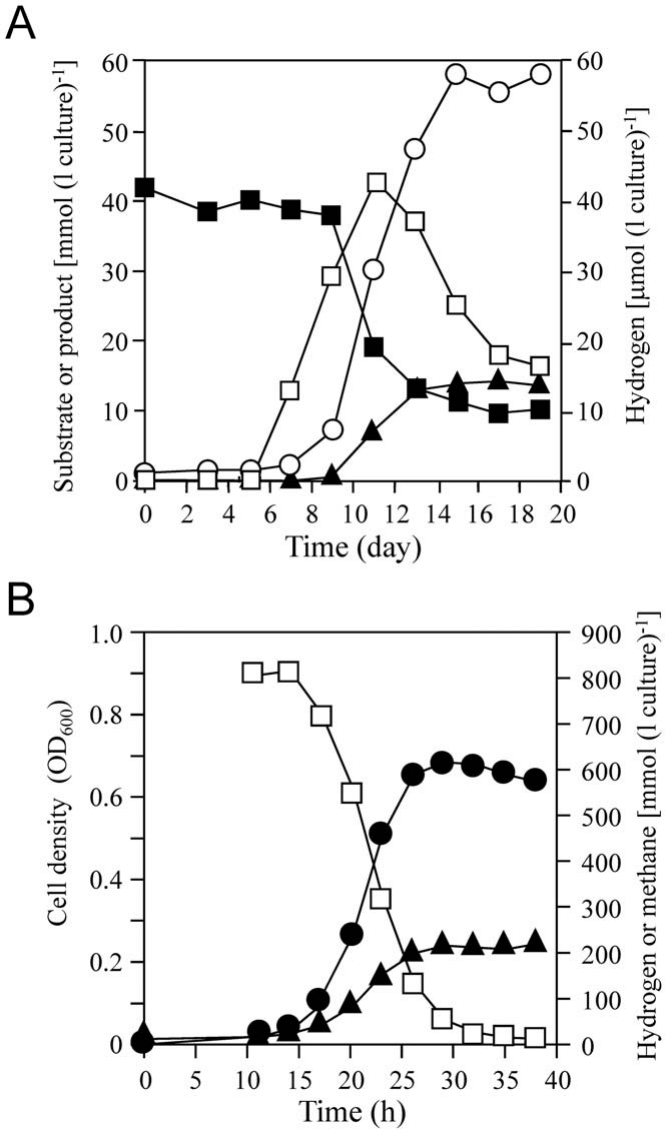
Growth of *M. thermautotrophicus* ΔH in co-culture with *S. lipocalidus* TGB-C1 and pure culture. (A) Time course of butyrate utilization (solid squares), methane (solid triangle), hydrogen (open squares), and acetate (open circle) production in a butyrate-oxidizing co-culture. The culture was grown in a 1.3-L serum bottle containing 500 ml of medium with 40 mM sodium butyrate as the substrate. (B) Time course of optical density (solid circle), hydrogen utilization (open squares), and methane production (solid triangle) in *M. thermautotrophicus* DH pure culture. The culture was grown in a 1.3-L serum bottle containing 100 ml of medium with H_2_ plus CO_2_ as the substrate.

Fig. 1A shows the time course of butyrate, acetate, methane, and H_2_ concentrations in a 500 ml-scale coculture. Butyrate consumption began after 9 days of inoculation, and 42 mM of initial substrate decreased to 10 mM in another 8 days. Although methane formation from butyrate was suspended after decreasing the substrate concentration to approximately 12 mM, this coculture finally, almost stoichiometrically, converted 32 mM of butyrate to 59 mM of acetate and 14 mM of methane (2CH_3_CH_2_CH_2_COOH+CO_2_+2H_2_O = 4CH_3_COOH+CH_4_). Only a trace amount of H_2_ (<43 µmol l^−1^ headspace) was detected during butyrate oxidation. This coculture is known to oxidize butyrate completely under aggregated condition [9]. Although aggregations consisting of *S. lipocalidaus* and *M. thermautotrophicus* were also found in our coculture (data not shown), it might be disturbed when culture sampling for monitoring substrate consumption, which might result in incomplete butyrate oxidation observed. The mean generation time in logarithmic growth phase calculated from methane formation was approximately 19 h. After half of the butyrate (20 mM) was consumed, which corresponds to logarithmic growth phase, the cells of *M. thermautotrophicus* were separated from syntroph cells by Percoll gradient centrifugation and subjected to proteomic analysis.

As a reference, pure cultured *M. thermautotrophicus* cells grown with abundant H_2_ were prepared by batch culture filled with 2 atm of H_2_ plus CO_2_ (80∶20), which is a standard concentration for ordinary laboratory cultures. The time course of cell density (OD at 600 nm), H_2_-utilization, and methane production in 100 ml-scale pure culture are shown in Fig. 1B. After approximately 10 h of lag phase, methanogen began to grow rapidly, in which the doubling time of *M. thermautotrophicus* estimated from methane formation was approximately 2.8 h. This indicated that the cells in pure culture grew 6.8 times faster than those in coculture. The approximately 800 mmol l^−1^ headspace of initial H_2_ was reduced to below 0.8 mmol l^−1^ headspace after 39 h of cultivation, and the final methane concentration reached 208 mmol l^−1^ headspace, which was consistent with the theoretical conversion rate of methane production from H_2_ plus CO_2_ (4H_2_+CO_2_ = CH_4_+2H_2_O). For sample preparation, methanogen cells were harvested at the early log phase to prevent H_2_ limitation.

### Proteome analysis

**Figure 2:**
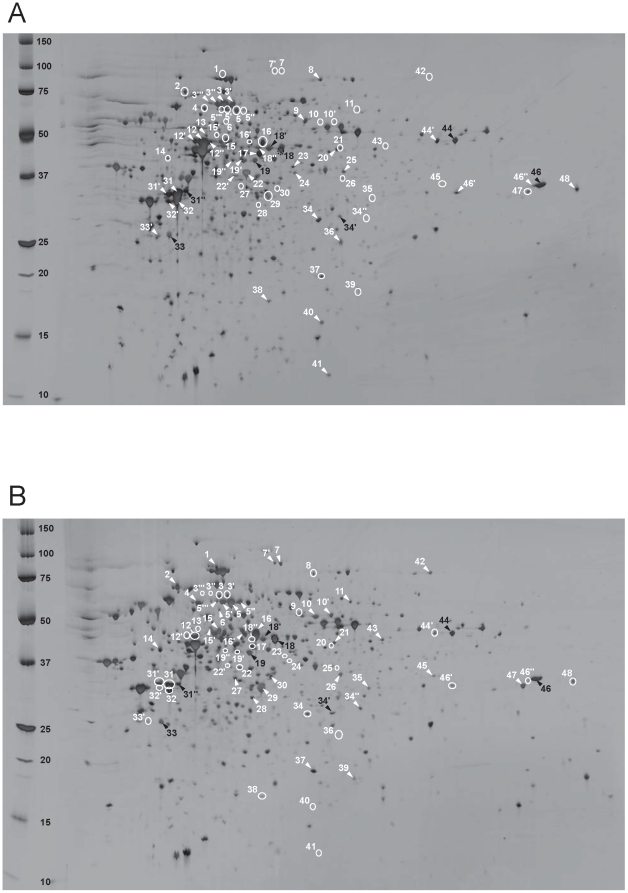
Comparative 2-DE analysis on pH range 4–7 of the proteins prepared from *M. thermautotrophicus* ΔH cells from (A) syntrophic co-culture and (B) pure-culture. Crude protein extracts (40 mg) were separated on Immobiline dry strips pH 4–7 and SDS-PAGE on 12–14% polyacrylamide, and detected by silver staining. The peptide spots indicated by white arrows and white circles appeared with higher and lower intensity, respectively, than the corresponding peptide spots of the other culture condition. The black-numbered spots exhibited a similar expression level in both conditions.

**Figure 3:**
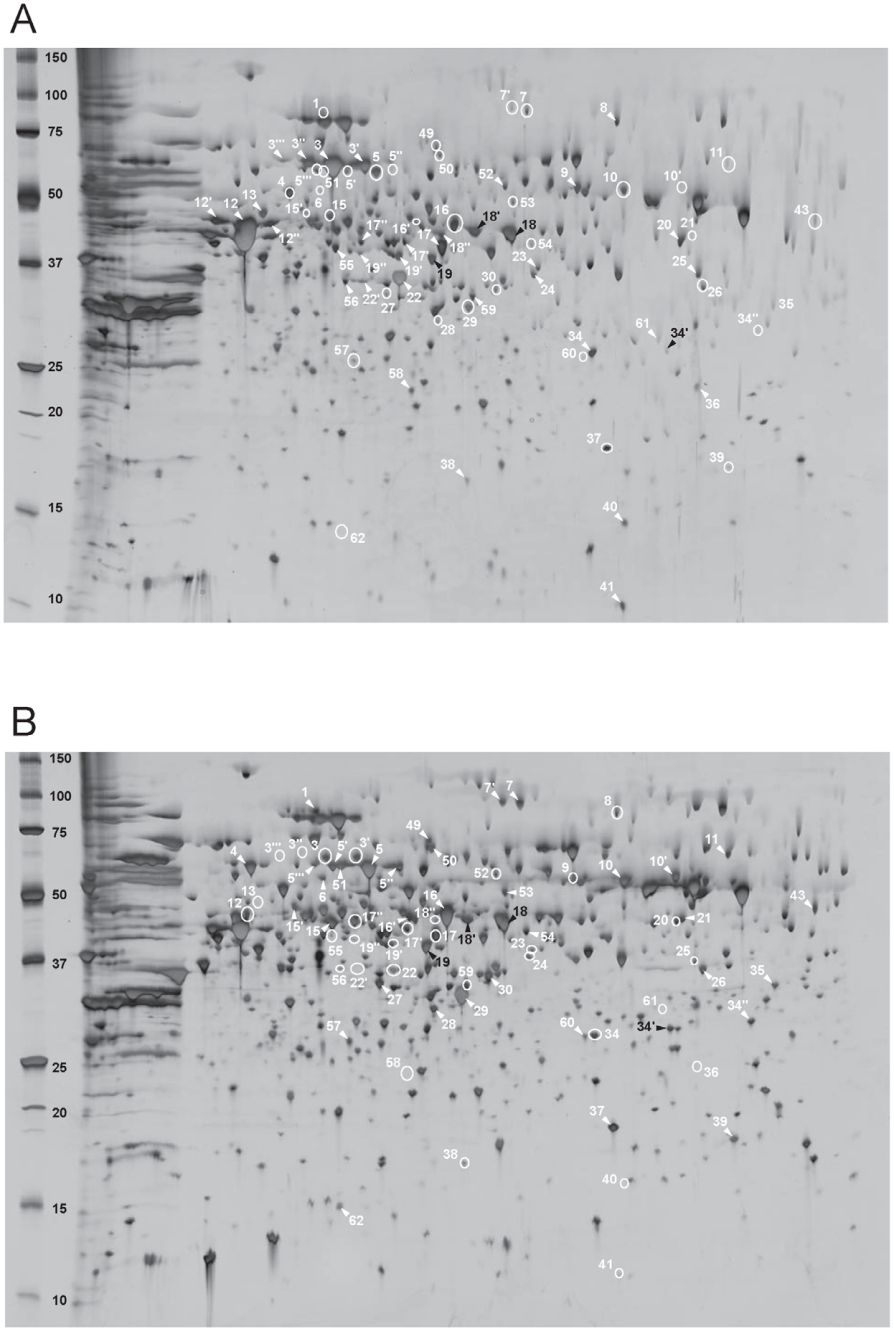
Two-DE analysis focused on the protein dense area (pH range 4.5–5.5) in **Fig. 2**. Proteins were prepared from *M. thermautotrophicus* ΔH in (A) syntrophic coculture and (B) pure culture. Crude protein extracts (60 mg) were separated on Immobiline dry strips pH 4.5–5.5 and SDS-PAGE on 12–14% polyacrylamide, and detected by silver staining. The peptide spots indicated by white arrows and white circles appeared with higher and lower intensity, respectively, than the corresponding peptide spots of the other culture condition. The black-numbered spots exhibited a similar expression level in both conditions.

Whole proteins extracted from *M. thermautotrophicus* cells grown in pure culture and in coculture with *S. lipocalidus* were subjected to comparative proteomic analysis using 2-DE. Preliminary experiments covering pH 3–10 demonstrated that nearly all proteins have acidic isoelectric points (pIs) below pH 7, which coincided with theoretical *M. thermautotrophicus* ΔH proteome (data not shown). Therefore, our proteome analysis focused on pH 4–7 (Fig. 2), and the protein-dense area was further separated at pH 4.5–5.5 (Fig. 3).

Silver-stained gel images obtained from triplicate cultivations for each culture condition were processed by 2-DE analysis software. The resulting 2-DE patterns at pH 4–7 and 4.5–5.5 are shown in Figs. 2 and 3, respectively. In the gels for pH 4–7, a total of 955 and 923 spots were detected in the gels for cocultured cells and pure cultured cells, respectively. In contrast, 632 and 599 spots were detected in the gels for pH 4.5–5.5 prepared from cocultured cells and pure cultured cells, respectively, in which 178 spots were detected with at least a two-fold intensity difference (p<0.05) (data not shown). In the present study, only conspicuous spots (159 spots at pH 4–7 and 112 spots at pH 4.5–5.5) were subjected to protein identification by peptide mass fingerprinting (PMF) using a matrix-assisted laser desorption/ionization time-of-flight mass spectrometer (MALDI-TOF/MS). Of the spots analyzed, 55 cytosolic proteins were differentially expressed between the two culture conditions, and these data are summarized in Table S1 according to functional categorization based on the KEGG (Kyoto Encyclopedia of Genes and Genomes) database (http://www.genome.ad.jp/kegg/).

### Methane genes

In our proteome analysis, major conspicuous protein spots were affiliated to methanogenesis-related proteins. Comparative analysis of detected spots showed that the expression levels of some methanogenesis-related proteins were largely different between the two culture conditions, and the expression pattern depended on the proteins. Particularly in the two MCR isozymes MCRI and MCRII, which catalyze the last step of methane-forming reactions, three major subunits, namely, α, β, and γ, showed the most significant change in spot intensity depending on the culture conditions examined (Figs. 2, 3, and Table S1). MCRI subunits were observed with remarkably large spots in the gels with coculture conditions, while they could be detected only as small spots in the gels for pure cultured cells (e.g., spots 3, 12, and 22 for MCRI α, β, and γ, respectively). On the other hand, the corresponding subunits of MCRII were observed to be abundant in pure cultured cells (e.g., spots 5, 16, and 29 for MCRII α, β, and γ, respectively). These results clearly indicated the preferential use of MCRI under coculture conditions, which is consistent with the previous observation for the gene and protein expressions of *M. thermautotrophicus* TM grown with acetate-oxidizing *T. phaeum* [2].

In addition to MCRs, proteins involved in cofactor F_420_-dependent reactions, such as F_420_-dependent *N* ^5^, *N* ^10^-methylene tetrahydromethanopterin dehydrogenase (MTD; spot 32), and F_420_-dependent *N* ^5^, *N* ^10^-methylene tetrahydromethanopterin reductase (MER; spots 31 and 31′), were also found to be expressed more in cocultured cells. On the other hand, tungsten formylmethanofuran dehydrogenase (FWD) subunit B (spots 10 and 10′) and some homologs of methanogenesis-related proteins whose functions are unclear, such as FWD C homolog (spot 60), F_420_-reducing hydrogenase (FRH) β subunit homolog (spot 42), and H_2_-dependent *N* ^5^, *N* ^10^-methylene tetrahydromethanopterin dehydrogenase homolog III (HMDIII; spot 14), were detected as large spots on the gels for pure cultured cells. These observations are likely due to the different H_2_ availability in the two culture conditions, since H_2_ supply could be the most significant factor for expression control of the genes responsible for F_420_-dependent reactions and HMDIII [10], [3].

### Genes other than methanogenesis

Of 55 differentially expressed proteins, 38 proteins were not directly involved in the methanogenesis pathway, and their deduced functions were distributed over a variety of categories as listed in Table S1. However, the most remarkable difference in protein expression was found in the enzymes responsible for carbohydrate and amino acid biosyntheses. The spot intensity of some key enzymes of the reductive TCA cycle, such as acetyl-CoA decarboxylase/synthase α (spots 7 and 7′) and β (spot 2) subunits (carbon monoxide dehydrogenase), pyruvate oxidoreductase β (spot 47) and γ (spot 37) subunits, phosphoenolpyruvate synthase (spot 1), and fumarate hydratase (spot 28), decreased in cocultured cells, suggesting the narrowed carbon fixation pathway and biosynthesis of derivative carbohydrates in syntrophically grown cells. In addition, smaller protein spots of indolepyruvate oxidoreductase (spot 49), glutamate synthase (spot 11), and aspartokinase (spot 54) indicated that some amino acid biosynthesis was repressed in cocultured cells. Moreover, translation activity of the methanogen cells might be controlled in response to culture conditions because the protein expression of RNA polymerase (B′ subunit; spot 50) and the translation initiation factor (IF2; spot 27) appeared to be downregulated. In addition, superoxide dismutase (spot 36) was found as a relatively large protein spot in coculture conditions, implicating oxidative stress response [11].

### Proteasomes and their modifications

During protein identification, some protein spots that were detected with similar molecular mass but different pI values were assigned to the same protein that seemed to indicate their isoforms (Table S1). Protein isoforms accompanied by horizontal migration might be the result of folding artifacts during IEF, or alternatively, of post- or cotranslational modifications. Some of the isoforms from FRH α, β, and γ (spots 44′, 46′-46″, and 33′, respectively), MER (spots 31 and 31′), flavoprotein AI (spot 18″), proteasome α (spots 34′ and 34″), and putative archaeal fructose 1.6-bisphosphatase (spots 19′ and 19″) changed their expression level depending on culture conditions.

**Figure 4:**
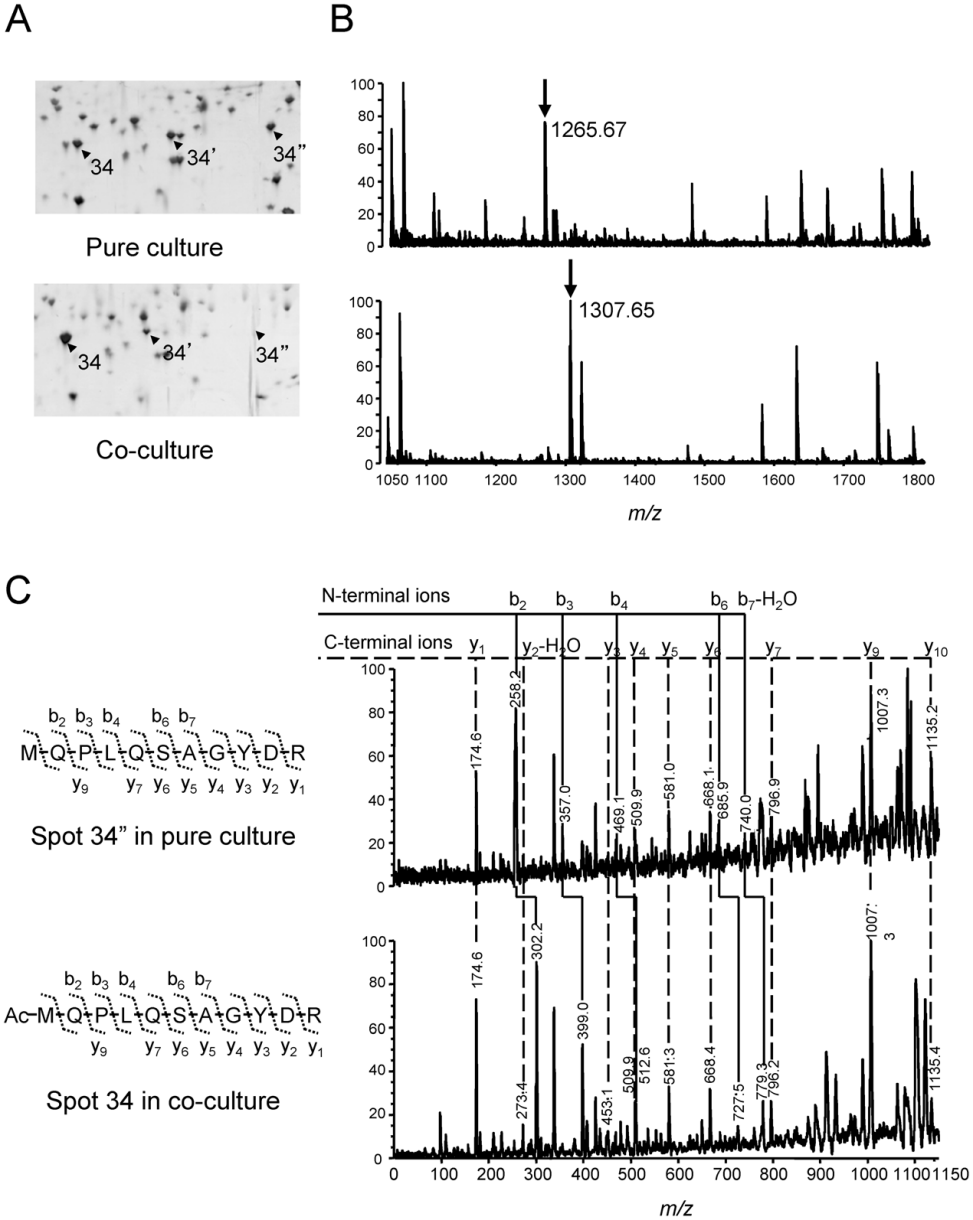
Two-DE and MALDI-TOF MS analyses of N-acetylation of proteasome α subunit. (A) Series of proteasome α subunits that appeared at different pI positions in 2-DE gels of pH 4.5–5.5. Both peptides from spots 34″ and 34 were identified as proteasome α. (B) Peptide mass of spots 34″ and 34 deduced by MALDI-TOF MS. The two different mass peaks at *m/z* 1265.61 and 1307.63 correspond to the polypeptides MQPLQSAGYDRA and Ac-MQPLQSAGYDRA, respectively. (C) MS/MS spectra of the N-terminal peptides obtained from spots 34″ (upper) and 34 (lower).

Of these proteins, isoforms of proteasome α were subjected to further evaluation for post- and/or cotranslational modifications. Proteasome α encoded by MTH686 was found to be differentially expressed between the two culture conditions, indicating that while three isoforms (spots, 34-34″) were detected in pure cultured cells, one of them, the most basic peptide (spot 34″), was not detected in coculture conditions. In addition, the most acidic isoform was slightly upregulated in cocultures (spot 34, Fig. 4A). All the isoforms showed obviously different pI values without significant molecular mass changes, indicating the possibility of post- and/or cotranslational modifications.

Proteasome is a proteolytic nanomachine shared among all domains of life, and archaeal proteasome resembles the eukaryal 20 S proteasome, which is a core particle of 26 S proteasome [12], [13]. Post- and co-translational modifications of the eukaryal and archaeal 20 S proteasomes have been reported [14], [15]; therefore, to obtain evidence of protein modifications, mass spectra that originated from each isoform were examined (Fig. 4B). The expected peak at *m/z* 1265.608, corresponding to the N-terminal peptide (MQPLQSAGYDR) of proteasome α, was not detected from these isoforms, except the most basic one derived from pure cultured cells. However, the *m/z* of N-terminal peptides of the remaining two acidic isoforms was found to change to 1307.619 with 42 Da of mass increase, which agreed with N-acetylated terminal peptides (Ac-MQPLQSAGYDR). Furthermore, MALDI collision-induced dissociation (MALDI-CID) analysis at *m/z* 1307.619 was performed to obtain sequence information for the isoforms (Fig. 4C). MS/MS spectra derived from spots 34 and 34′ proved the presence of the acetyl group at N-termini of these isoforms. These observations suggested that N-termini of proteasome α were acetylated in cocultured cells.

## Discussion

To better understand the physiological status of hydrogenotrophic methanogens living on interspecies H_2_ transfer, we constructed a syntrophic coculture consisting of *M. thermautotrophicus* and butyrate-oxidizing *S. lipocalidus*, and the protein expression of the methanogens was compared by proteome analysis to that of pure cultured cells. This comparative proteome analysis showed that global protein expression of methanogen cells in the model coculture was substantially different from that of pure cultured cells. In brief, in syntrophic coculture conditions, although methanogenesis-driven energy generation appeared to be maintained by shifting the pathway to alternative MCRI and F_420_-dependent processes, the proteins involved in cellular component biosynthesis or metabolism tended to be downregulated, suggesting restrained cell growth rather than vigorous proliferation. Such a characteristic protein expression pattern of cocultured cells was considered to represent the unique cellular physiology of the methanogens living on interspecies H_2_ transfer.

In the present study, many conspicuous protein spots were affiliated with methanogenesis-related proteins because of their unusual abundance in total proteins. Of these proteins, the most striking difference in protein expression was found in MCR isozymes between the two culture conditions, in which MCRI was preferentially used in cocultured cells in contrast to the predominant expression of MCRII in pure cultures. These results are basically consistent with those of our previous report using acetate-oxidizing *T. phaeum* as a syntrophic partner [2]. Since gene and protein expressions of MCRs were strictly regulated by H_2_ availability [16], [17], the preferential use of MCRI likely indicates the limited H_2_ supply to the methanogen cells grown on interspecies H_2_ transfer. Hydrogen partial pressure needed for anaerobic butyrate oxidation has been estimated to be approximately 100 Pa [18]. Although it is higher than that found in acetate-oxidizing coculture using *T. phaeum* (approximately 50 Pa), it is still extremely low compared to the H_2_ concentrations normally used for pure cultures (10^5^ Pa). These observations suggest that preferential use of MCRI is a general physiological feature shared among *M. thermautotrophicus* cells grown with syntrophic bacteria.

In addition to MCRI, some proteins responsible for F_420_-dependent reactions in the methanogenesis pathway, such as MTD and MER, were also found to be highly expressed in cocultured cells. The cofactor F_420_ is known to serve as an electron carrier in the methanogenesis pathway, and the robust expression of methane genes related to F_420_-dependent processes is a general cell response of hydrogenotrophic methanogens observed in H_2_-limited culture conditions [19], [3]. The expression pattern of methanogenesis-related proteins in our study suggested that H_2_ availability is the most significant factor affecting the physiological features of methanogens in syntrophic associations.

The proteome analysis, coupled with silver staining, considerably increased detectable proteins compared with a previous study that used Coomassie brilliant blue-stained gel [2]. This allowed a comprehensive evaluation of the global protein expression of this methanogen. As a result, a number of proteins belonging to various functional categories were found to be differentially expressed between the two culture conditions, together with methanogenesis-related proteins. Based on the observation that some key enzymes related to biosynthesis and metabolism of cellular components were downregulated in coculture conditions, active proliferation of the methanogen cells was considered to be strictly repressed under syntrophic growth conditions.

However, strong expression of MCRI subunits and other methanogenesis-related enzymes, such as FRH, MTD, and MER, suggested that MCRI-based methanogenesis and energy generation via proton motive force were maintained even under H_2_-limited syntrophic conditions. Such a characteristic protein expression pattern has also been reported from methanogen cells cultivated at low temperature. Goodchild *et al.* [20] conducted a comparative proteomic analysis of the antarctic methanogen *Methanococcoides burtonii* grown at low and optimum temperature (4°C and 23°C), and showed that although the proteins related to cellular component biosynthesis were equally repressed, methanogenesis-responsible enzymes were consistently expressed even at 4°C. This occurred to satisfy a minimum energy demand, even under such unfavorable growth conditions. Although such metabolic change could lead to constant methane production without increasing biomass in methanogenic ecosystems, the repression of biosynthesis and energy consumption, which was suggested by down-regulation of the relevant genes, would help methanogens survive in situations unsuitable for their growth, which is thought to be one strategy to thrive in H_2_-limited natural environments.

Another interesting finding is the suggestive evidence for culture condition-dependent post- and/or cotranslational modifications on α subunits of proteasome, which implies that the control of enzyme activity by protein modifications, together with the regulation of protein expression, may contribute to the physiological adaptation of this methanogen to different culture conditions. The proteasome is an enormous protein complex responsible for intracellular protein degradation and turnover, and it controls several pivotal cellular events such as cell cycle, stress response, and transcriptional regulation [21]. Archaeal proteasome corresponds to 20 S proteolytic component of eukaryal 26 S proteasome, forming a cylindrical shape of four-striped side-views and sevenfold-symmetric top-views (α_7_β_7_β_7_α_7_), which is a typical structure highly conserved from archaea to eukaryotes [22], [23]. An interior chamber of the cylinder is proteolytically active, and N-terminal tails of ring-forming α subunits are evidently important to its gate structure [24], [25], [26].

The gating control of the proteasome has been predicted to be regulated by N-terminal post- and/or cotranslational modifications on the basis of the observation that the lack of N-acetylation on α subunits of yeast proteasome has enhanced proteolytic activity [15]. In the present study, non-acetylated isoforms of proteasome α (spot 34″) were detected only in pure cultured cells, while acetylated isoforms increased in syntrophically grown cells (spot 34). On the basis of reports of yeast proteasome, the observed modification pattern of proteasome might indicate higher proteolytic activity of pure cultured cells than that of cocultured cells, which implies repressed protein turnover in cocultured cells. However, our data also suggest the possibility of additional modification(s) to proteasome α, since two isoforms (spots 34 and 34′) with acetylated N-termini appeared at different pI positions. Although phosphorylation is known as another general modification of proteasome subunits [27], [28], [29], no certain evidence was obtained in this study.

To our knowledge, the present study is the first report of N-acetylation of proteasome subunits in methanogenic archaea, although it has been found in haloarchaea [14]. N-acetylation in archaeal proteins generally occurs on small amino acid residues such as serine or alanine after removal of the first methionine residue. However, N-acetylated isoforms of proteasome α observed in this study retain the first methionine residue. Direct acetylation of methionine residue was also found in some proteins in archaea, including proteasome α, which suggests activity control by N-acetylation and the involvement of yet uncharacterized acetyltransferase for the modification [30], [14].

## Materials and Methods

### Microbes, media, and growth conditions

*M. thermautotrophicus* strain ΔH (DSM1053) was purchased from Deutsche Sammlung von Mikroorganismen und Zellkulturen GmbH (Braunschweig, Germany). The thermophilic butyrate-oxidizing bacterium *Syntrophothermus lipocalidus* strain TGB-C1 (DSM12680) in coculture with *M. thermautotrophicus* was a gift from Yuji Sekiguchi.

*M. thermautotrophicus* in pure culture was grown in a 1.3-L serum bottle containing 100 ml of minimal salts medium, as described previously [31] except that pH was not adjusted, under an atmosphere of H_2_-CO_2_ (80∶20 [v/v]) at 2 atm. The medium was reduced by adding 1% (v/v) of 30 g L^−1^ Na_2_S-9H_2_O and cysteine-HCl. All cultivations were carried out at 55°C and stirred with a 40-mm teflon-coated stirrer bar at 700 rpm. A syntrophic coculture consisting of *M. thermautotrophicus* and *S. lipocalidus* was grown in a 1.3-L serum bottle containing 500 ml of the medium, as described above, with 40 mM of sodium butyrate under an atmosphere of N_2_-CO_2_ (80∶20 [v/v]) at 1 atm. All cultures were grown at 55°C without either stirring or shaking. Samples for 2-DE were prepared from three independent growth experiments for each culture condition.

### Measurements of butyrate, acetate, methane, and hydrogen

The methane and hydrogen in the headspace were determined using a gas chromatograph (GC8-AIT, Shimadzu, Kyoto, Japan) equipped with a 60/80-mesh column (Unibeads C, Shimadzu) and a thermal conductivity detector. Argon was used as a carrier gas. The column and the detector temperatures were kept at 145°C and 150°C, respectively. Butyrate and acetate in the medium were measured with a high-performance liquid chromatograph (SCL-6A, Shimadzu) fitted with an ion exclusion column (Shim-pack 101H, Shimadzu) and a UV detector (SPD-6AV, Shimadzu). The column was operated at 40°C, and 17 mM HClO_4_ was used as a carrier at a flow rate of 1 ml min^−1^. The sample of medium was filtrated with a membrane filter with 0.22-µm pore size before measurements.

### Percoll purification of methanogen cells

*M. thermautotrophicus* cells were purified from syntrophic butyrate-oxidizing coculture by density gradient centrifugation according to the procedure previously described [2]. The cells of the syntrophic butyrate-oxidizing coculture were harvested at the point of 50% substrate consumption, and the methanogen cells and syntroph cells were fractionated by Percoll-gradient centrifugation. The coculture cells were harvested by centrifugation at 10,000× *g* and 4°C for 15 min, washed with 10 mM Tris-HCl buffer (pH 8.0), and resuspended in 0.5 ml of the same buffer. Percoll (GE Healthcare, Little Chalfont, Buckinghamshire, UK) and 2.5 M sucrose solution were anaerobically prepared by bubbling with N_2_ gas before autoclaving, and then mixed at a ratio of 9∶1 (Percoll : 2.5 M sucrose solution) to give a final density of approximately 340 mosmol kg^−1^ of H_2_O. The cell suspension was loaded onto 37.5 ml of Percoll-sucrose solution inside an anaerobic chamber (MACS Anaerobic Workstation, Don Whitley Scientific, UK). The density gradient was self-generated and yielded two distinct bands by centrifugation at 45,000× g for 3 h by using an ultracentrifuge (L-70 fitted with a type 70 Ti rotor, Beckman Coulter, Fullerton, CA, USA). The band with higher density (the lower band), which was mostly comprised *M. thermautotrophicus* cells, was carefully taken with a sterile syringe, and Percoll particles were subsequently removed by washing with the same buffer as that used above. The cells were then stored at −80°C until use.

### Sample preparation

Methanogen cells were harvested in logarithmic growth phase from pure cultures and from those fractionated from cocultures with the butyrate-oxidizing bacterium, as described above. The cell pellets were resuspended in 10 mM Tris-HCl (pH 8.0) containing 5 µg ml^−1^ DNase I, 10 µg ml^−1^ RNase A and 1% (v/v) protease inhibitors (Protease Inhibitor Mixture for Bacterial Cell Extracts, Wako Pure Chemical, Osaka, Japan) to a target ratio of 100 mg of wet weight ml^−1^. Cell suspensions were transferred into a 2-ml microtube containing 0.2 g of glass beads (0.1 mm in diameter; BioSpec, Bartlesville, OK, USA) and then disrupted with a bead-beating device (FastPrep model FP120, Qbiogene, Morgan Irvine, CA, USA) at a speed of 6.0 for 3 min. The microtube was cooled on ice for 30 s every 30 s of the process. The resultant lysate was centrifuged at 16,000× *g* for 10 min at 4°C, and the supernatant was collected. The pellet of unbroken cells was resuspended in 800 µl of a solubilization buffer containing 8.5 M urea, 4% (w/v) 3-[(3-cholamidopropyl) dimethylammonio]-1-propanesulfonate (CHAPS), 2% (v/v) IPG buffer (pH 4–7 or 4.5–5.5; GE healthcare), 1% (w/v) dithiothreitol (DTT), and 1% (v/v) protease inhibitor (described above) and ruptured by bead-beating for 1 min on ice. The resultant lysate was centrifuged at 16,000× *g* for 10 min at 4°C, and the supernatant was collected. Both supernatants were combined and transferred into a polyallomer centrifuge tube (13×51 mm; Beckman Coulter) and centrifuged at 200,000× *g* for 15 min, at 4°C using a TLA100.3 rotor (Beckman Coulter). The supernatant was stored at −80°C before use. The protein concentration was measured by modified Bradford method with the Protein assay kit using IgG as the standard (both Bio-Rad, Hercules, CA, USA).

### Two-dimensional gel electrophoresis and image analysis

2-DE separation of proteins from *M. thermautotrophicus* was performed using a Multiphor II gel electrophoresis apparatus (GE Healthcare). The protein extract was diluted with a rehydration buffer containing 7 M urea, 2 M thiourea, 4% CHAPS, 13 mM DTT, 0.5% IPG buffer (pH 4–7 or pH 4.5–5.5, GE Healthcare), and 0.01% bromophenol blue (BPB) to give a final volume of 340 µl. Isoelectric focusing (IEF) was performed with two different pH gradients of 18 cm IPG strips (Immobiline DryStrips, pH 4–7 or pH 4.5–5.5, GE Healthcare). The pH 4–7 strip and pH 4.5–5.5 strip were rehydrated with rehydration buffer that included 40 µg and 60 µg of protein, respectively, prepared as described above for 16 h at ambient temperature.

IEF was carried out at 20°C using the gradient mode with total focusing of 59 kVh. After IEF, the IPG strip was equilibrated in 20 ml of equilibration buffer containing 50 mM Tris-HCl (pH 8.8), 6 M urea, 30% (w/v) glycerol, 2% (w/v) SDS, and 1.2% (w/v) DTT, followed by a second equilibration step replacing DTT with 3% iodoacetamide (IAA) and adding 0.01% BPB. SDS-PAGE was performed with a precast 12–14% polyacrylamide gradient gel (Excel-Gel™XLSDS 12–14, GE Healthcare) according to the manufacturer's directions. The Precision Plus Protein Standards (Bio-Rad) were used as a size standard. The gels were stained by the silver-staining method with the PlusOne Silver Staining Kit, Protein (GE Healthcare) according to the procedures modified for the subsequent PMF analysis using MALDI-TOF MS [32]. The stained gels were imaged using a color image scanner (ES-2200, EPSON, Suwa, Japan), and the raw scans were processed and analyzed using the 2-DE analysis software PDQuest (Bio-Rad).

The triplicate gel images, which were run in parallel to the three individual cultivation samples for each condition, were edited and matched to each other, and then subjected to quantitative analysis. Student's t-test (95% confidence interval) was performed to determine the significance of spot intensity differences, and at least two-fold changes in spot intensity were considered.

### Mass spectrometric identification of proteins

Tryptic in-gel digestion of the spots of interest was carried out according to the same procedure reported by Katayama *et al.* [33]. The spots were excised and destained using a mixture of 15 mM potassium ferricyanide and 50 mM sodium thiosulfate with vigorous shaking. The gel pieces were vigorously washed several times with 50% methanol/10% acetic acid. After washing, the gel pieces were swollen in 100 mM ammonium bicarbonate, then shaken with 100% acetonitrile, and completely dried with the SpeedVac evaporator (CVE-100D, EYELA, Tokyo, Japan). The gel pieces were swollen in 0.1 µg µl^−1^ modified trypsin (Promega, Southampton, UK) in 100 mM ammonium bicarbonate containing 0.1% n-octyl-β-d-glucoside (Dojindo, Kumamoto, Japan) and incubated on ice for 40 min, which was followed by further incubation at 37°C for 4 h. The tryptic peptides were extracted with 75% acetonitrile in 0.1% trifluoroacetic acid (TFA). The extracts were concentrated with the SpeedVac.

The resulting solution was subjected to MALDI-TOF-MS analysis. A saturated solution of 4-hydroxy-α-cyanocinnamic acid (CHCA) in 50% acetonitrile/0.1% TFA and a solution of 2,5-dihydroxybenzoic acid (DHB) in 33% acetonitrile/0.1% TFA (40 mg ml^−1^) were prepared as the matrix solution. Either CHCA or DHB solution was mixed with equal volume (0.5 µl) of the peptides solution and spotted onto the MALDI target plate. MALDI-TOF-MS measurements were carried out with a Voyager DE-PRO time-of-flight mass spectrometer (Applied Biosystems, Framingham, MA, USA). The mass spectral data were obtained using a positive reflectron mode in range of m/z 800–3500 and internally calibrated using trypsin autolysis peaks (*m/z* 842.5099, 2211.1045 and 2807.3145).

The peptide mass list was searched with the Mascot (Matrix Science, Boston, MA, USA) database search engine (http://www.matrixscience.com/) [34] against the NCBI nonredundant (nr) database and the Swiss-Prot protein database. Database searches were performed with the following parameters: 50 ppm mass tolerance (70 ppm for low resolution samples), 1 missed cleavage, carbamidomethylation of cysteine as the fixed modification, and oxidation of methionine as the variable modification and species were limited to archaea. Protein identification was judged on its significance score p<0.05 and the accuracy of its taxonomic name, *M. thermautotrophicus* strain ΔH.

Sequence information of N-terminal modifications of proteasome was obtained by peptide fragmentation by MALDI collision-induced dissociation (CID) on AXIMA CFR-plus time-of-flight mass spectrometer (Shimadzu/Kratos, Kyoto, Japan). Formed fragment masses were compared with theoretical masses via the MS-Product program of Proteinprospector (http://prospector.ucsf.edu/).

## Supporting Information

Table S1 List of proteins differentially expressed between pure-cultures and co-cultures.
(DOC)
