# Supplementary material for: Comparative Proteomic Analysis of Methanothermobacter themautotrophicus ΔH in Pure Culture and in Co-Culture with a Butyrate-Oxidizing Bacterium
Source: PLoS One. 2011 Aug 31;6(8):e24309. doi: 10.1371/journal.pone.0024309 (PMC3164167; doi:10.1371/journal.pone.0024309)
Supplement: Table S1 — List of proteins differentially expressed between pure-cultures and co-cultures. (DOC) [file pone.0024309.s001.doc]

Table S1. List of proteins differentially expressed between pure-cultures and co-cultures.

| Spot no. in | Spot no. in | MTH | Protein name*^b^* | Category*^c^* | Theoretical | Expression*^e^* |
| --- | --- | --- | --- | --- | --- | --- |
| pH4-7*^a^* | pH4.5-5.5*^a^* |  |  |  | pI/MW(kDa)*^d^* |  |
| 3-3´´´, 13, 25, | 3-3´´´, 13, 25, | 1164 | Methyl coenzyme M reductase I, α subunit | A | 4.82 / 60.48 | **↑** |
| 40 | 40, 61 |  |  |  |  |  |
| 12-12´´ | 55, 58 | 1168 | Methyl coenzyme M reductase I, β subunit | A | 4.76 / 48.14 | **↑** |
| 22, 22´ | 22, 22´ | 1165 | Methyl coenzyme M reductase I, γ subunit | A | 4.82 / 29.10 | **↑** |
| 5-5´´´ | 5-5´´´ | 1129 | Methyl coenzyme M reductase II, α subunit | A | 4.88 / 60.71 | ↓ |
| 16, 16´ | 16, 16´ | 1132 | Methyl coenzyme M reductase II, β subunit | A | 4.93 / 47.48 | ↓ |
| 29 | 29 | 1130 | Methyl coenzyme M reductase II, γ subunit | A | 4.95 / 30.55 | ↓ |
| (44), 44´ |  | 1300 | F_420_-reducing hydrogenase, α subunit | A | 5.58 / 45.17 | **↑** |
| (46), 46´, 46´´ |  | 1297 | F_420_-reducing hydrogenase, β subunit | A | 6.07 / 30.77 | **↑** |
| (33), 33´ |  | 1298 | F_420_-reducing hydrogenase, γ subunit | A | 4.69 / 30.21 | **↑** |
| 42 |  | 280 | F_420_-reducing hydrogenase, β subunit homolog | A | 5.58 / 76.66 | **↓** |
| 32, 32´ |  | 1464 | F_420_-dependent *N*^5^, *N*^10^-methylene tetrahydromethanopterin | A | 4.60 / 29.60 | **↑** |
|  |  |  | dehydrogenase |  |  |  |
| 14 |  | 504 | H_2_-dependent *N*^5^, *N*^10^-methylene tetrahydromethanopterin | A | 4.61 / 37.53 | ↓ |
|  |  |  | dehydrogenase (III) |  |  |  |
| 10, 10´ | 10, 10´ | 1559 | Tungsten formylmethanofuran dehydrogenase subunit B | A | 5.24 / 48.89 | **↓** |
|  | 60 | 192 | Tungsten formylmethanofuran dehydrogenase subunit C homolog | A | 5.04 / 24.49 | **↓** |
| 39 | 39 | 1714 | Formate hydrogenlyase, iron-sulfur subunit 2 | A | 5.21 / 38.39 | **↓** |
| 31, 31´, (31´´) |  | 1752 | F_420_-dependent *N*^5^, *N*^10^-methylene tetrahydromethanopterin | A | 4.59 / 33.48 | **↑** |
|  |  |  | reductase |  |  |  |
| 17 | 17, 17´, 17´´ | 1512 | H_2_-dependent *N*^5^, *N*^10^-methylene tetrahydromethanopterin | A | 4.91 / 37.30 | **↑** |
|  |  |  | dehydrogenase (II) |  |  |  |
| (18, 18´), 18´´ | (18, 18´), 18´´ | 1350 | Flavoprotein AI | B | 5.01 / 45.98 | **↑** |
| 20 | 20 | 220 | Flavoprotein A homolog (II) | B | 5.18 / 45.74 | **↑** |
| Table S1. Continued. | | | | | | |
| Spot no. in | Spot no. in | MTH | Protein name*^b^* | Category*^c^* | Theoretical | Expression*^e^* |
| pH4-7*^a^* | pH4.5-5.5*^a^* |  |  |  | pI/MW(kDa)*^d^* |  |
| 47 |  | 1738 | Pyruvate oxidoreductase, β subunit | B | 6.09 / 31.59 | **↓** |
| 37 | 37 | 1740 | Pyruvate oxidoreductase, γ subunit | B | 6.03 / 28.63 | **↓** |
|  | 49 | 1852 | Indolepyruvate oxidoreductase, α subunit | B | 4.92 / 66.87 | **↓** |
| 30 |  | 1511 | Arsenical pump-driving ATPase | C | 4.95 / 36.54 | ↓ |
| 28 | 28 | 1735 | Fumarate hydratase, class I | D | 4.87 / 30.25 | **↓** |
| 5´ | 51 | 701 | Acetyl-CoA synthetase related protein | E | 4.78 / 48.55 | ↓ |
| 1 | 1 | 1118 | Phosphoenolpyruvate synthase | E | 4.77 / 75.62 | **↓** |
| 7, 7´ | 7, 7´ | 1708 | Acetyl-CoA decarboxylase/synthase complex, α subunit | F | 4.99 / 86.07 | **↓** |
| 2 |  | 1710 | Acetyl-CoA decarboxylase/synthase complex, β subunit | F | 4.57 / 51.74 | **↓** |
| 21 | 21 | 1522 | Nitrogenase α chain (NifD) related protein | G | 5.23 / 39.10 | **↓** |
| 11 | 11 | 194 | Glutamate synthase (NADPH) | H | 5.35 / 54.01 | **↓** |
|  | 54 | 802 | Aspartokinase II, α subunit | H | 5.11 / 44.04 | **↓** |
| 24 | 24 | 119 | ATP phosphoribosyltransferase related protein | I | 5.22 / 37.43 | **↑** |
|  | 59 | 1223 | Inosine-5’-monophsphate dehydrogenase related protein II | J | 4.99 / 31.13 | **↑** |
|  | 56 | 1224 | Inosine-5’-monophsphate dehydrogenase related protein III | J | 4.85 / 34.26 | **↑** |
| 48 |  | 1225 | Inosine-5’-monophsphate dehydrogenase related protein IV | J | 6.05 / 29.95 | **↑** |
| 33 |  | 373 | dTDP-glucose 4,6-dehydrogenase related protein | K | 5.49 / 39.16 | **↓** |
| 15, 15´ | 15, 15´ | 1543 | Thiamine biosynthesis protein | L | 4.83 / 47.29 | ↓ |
| 23 | 23 | 792 | 3-hydroxy-3-methylglutaryl-CoA synthase | M | 5.15 / 37.08 | **↑** |
| 8 | 8 | 1639 | Cell division control protein Cdc48 | N | 5.15 / 80.60 | **↑** |
| 4 | 4 | 218 | Thermosome, α subunit | O | 4.73 / 60.43 | ↓ |
| 34´´ |  | 686 | Proteasome, α subunit | O | 5.30 / 27.53 | **↑** |
| (34), 34´ |  | 686 | Proteasome, α subunit | O | 5.30 / 27.53 | ↓ |
| 36 | 36 | 160 | Superoxide dismutase (Fe/Mn) | P | 5.26 / 24.10 | **↑** |
| Table S1. Continued. | | | | | | |
| Spot no. in | Spot no. in | MTH | Protein name*^b^* | Category*^c^* | Theoretical | Expression*^e^* |
| pH4-7*^a^* | pH4.5-5.5*^a^* |  |  |  | pI/MW(kDa)*^d^* |  |
| 45 |  | 1383 | DNA repair protein RadA | Q | 5.69 / 34.12 | **↓** |
|  | 50 | 1050 | DNA-dependent RNA polymerase, subunit B’ | R | 4.91 / 67.69 | **↓** |
| 27 |  | 1308 | Translation initiation factor IF-2, α subunit | S | 5.34 / 29.78 | **↓** |
|  | 57 | 1020 | IMP cyclohydrolase | T | 4.78 / 21.84 | **↓** |
| 6 | 6 | 1137 | Conserved protein (FlpA) | T | 4.82 / 55.11 | **↓** |
| 9 | 9 | 101 | Hypothetical protein | T | 5.11 / 56.13 | **↑** |
| 57 |  | 141 | Hypothetical protein | T | 4.86 / 25.09 | **↓** |
|  | 52 | 210 | Hypothetical protein (predicted ATPase with chaperone activity) | T | 5.01 / 59.20 | **↑** |
| 41 | 41 | 691 | Hypothetical protein | T | 5.20 / 14.01 | **↑** |
| 47 | 47 | 1008 | Hypothetical protein (type II topoisomerase VI, subunit A) | T | 5.39 / 40.03 | **↓** |
|  | 62 | 1351 | Hypothetical protein | T | 4.86 / 15.44 | **↓** |
|  | 53 | 1394 | Hypothetical protein | T | 5.00 / 46.56 | **↓** |
| 38 | 38 | 1410 | Hypothetical protein | T | 5.01 / 18.30 | **↑** |
| (19), 19´, 19´´ | (19), 19´, 19´´ | 1686 | Hypothetical protein (archaeal fluctose 1.6-bisphosphatase) | T | 4.94 / 40.29 | **↑** |

*^a^* Protein spots derived from same protein were given same number shared between the two gel images, and spot numbers correspond to those in Fig. 2 (pH 4-7) or 3 (pH 4.5-5.5). Spot numbers with prime symbiol represent the isoforms having different pI, and the number of primes correspond to intensity of spot in descending order. Spot numbers in brackets showed no significant spot intensity change between pure- and co-culture conditions.

*^b^*Protein names are of the result from Mascot serach via NCBI database. Protein names annotated by COG are also indicated in brackets.

*^c^*Functional categories are followed by KEGG database (http://www.genome.ad.jp/kegg/) and the abbreviations are as follows; A: hydrogen metabolism and methanogenesis; B: electron transport and redox metabolism; C: ATPases; D: citrate cycle; E: pyruvate and acetyl-CoA metabolism; F: carbon fixation; G: nitrogen metabolism; H: alaine, asparate, and glutamate metabolism; I: histidine metabolism; J: phenylalanine, tyrosine, and tryptophan metabolism; K: nucleotide sugar metabolism; L: cofactor metabolism; M: sterol metabolism; N: cell division; O: chaperones; P: detoxification; Q: DNA metabolism, modification, and replication; R: transcription and RNA processing; S: translation factors; T: unknown function.

*^d^*Computed by SwissProt from the genome sequence (<http://au.expasy.org/tools/pi_tool.html>).

*^e^*Expression change in co-culture condition comparing to pure-cultures is indicated by up- and down-arrows.
